# Biomarker-driven drug repurposing on biologically similar cancers with DNA-repair deficiencies

**DOI:** 10.3389/fgene.2022.1015531

**Published:** 2022-12-13

**Authors:** Seeya Awadhut Munj, Tasnimul Alam Taz, Suzan Arslanturk, Elisabeth I. Heath

**Affiliations:** ^1^ Department of Computer Science, Wayne State University, Detroit, MI, United States; ^2^ Department of Oncology, Wayne State University, Detroit, MI, United States; ^3^ Molecular Therapeutics Program, Barbara Ann Karmanos Cancer Institute, Detroit, MI, United States

**Keywords:** drug repurposing, DNA repair, personalized medicine, multi cancer treatment, mitoxantrone, homologous recombination

## Abstract

Similar molecular and genetic aberrations among diseases can lead to the discovery of jointly important treatment options across biologically similar diseases. Oncologists closely looked at several hormone-dependent cancers and identified remarkable pathological and molecular similarities in their DNA repair pathway abnormalities. Although deficiencies in Homologous Recombination (HR) pathway plays a significant role towards cancer progression, there could be other DNA-repair pathway deficiencies that requires careful investigation. In this paper, through a biomarker-driven drug repurposing model, we identified several potential drug candidates for breast and prostate cancer patients with DNA-repair deficiencies based on common specific biomarkers and irrespective of the organ the tumors originated from. Normalized discounted cumulative gain (NDCG) and sensitivity analysis were used to assess the performance of the drug repurposing model. Our results showed that Mitoxantrone and Genistein were among drugs with high therapeutic effects that significantly reverted the gene expression changes caused by the disease (FDR adjusted p-values for prostate cancer =1.225e-4 and 8.195e-8, respectively) for patients with deficiencies in their homologous recombination (HR) pathways. The proposed multi-cancer treatment framework, suitable for patients whose cancers had common specific biomarkers, has the potential to identify promising drug candidates by enriching the study population through the integration of multiple cancers and targeting patients who respond poorly to organ-specific treatments.

## 1 Introduction

Developing a new drug for a condition can take around 10–13 years and close to 2.8 billion dollars (DiMasi et al., 2016). Despite this, 90% of the drug candidates entering clinical trials fail (Sun et al., 2022). Human body is a complex system, with myriad interactions taking place simultaneously, interdependent on each other. The same pathway or mechanism involving certain genes, may be responsible for different diseases. A drug developed for a particular condition, therefore, could be a potential candidate for another condition. Drug repurposing can drastically reduce the time and cost of developing new drugs by searching for FDA-approved drugs, drugs under trial, or other chemicals that have a therapeutic effect on conditions outside the scope of the original medical indication (Pushpakom et al., 2019). Drug repurposing minimizes the chances of failure in clinical trials and reduces time for approval.

Similar molecular and genetic aberrations among diseases can lead to the discovery of jointly important treatment options across biologically similar diseases. Oncologists have closely looked at prostate, ovarian and breast cancers and identified that the tumors arising from these cancers are typically hormone-dependent and have remarkable underlying pathological and molecular similarities in their DNA repair pathway abnormalities (Risbridger et al., 2010). Analyzing patient data from biologically similar cancers together provides insights into their similarities as well as knowledge about individual cancers, which may not have been possible by analyzing individual cancer data separately. Zhou et al. (2021) identified jointly important biomarkers across breast, prostate and ovarian cancers by utilizing patient data from the three cancers using a cross-cancer learning approach. This reiterates that the same pathway or a gene is responsible for multiple diseases. These biological similarities have led to remarkably similar treatment options. For instance, combining the androgen deprivation therapy (ADT) with PARP inhibitors (i.e. drugs already used in breast cancer treatment) showed to be an effective approach in reducing the progression and recurrence of prostate cancer. Several single agent activity PARP inhibitors (PARPi) were recently approved for treating certain ovarian and breast cancers (Asim et al., 2017). The US Food and Drug Administration (FDA) approved the first multi-cancer treatment (Keytruda^®^), for patients whose cancers had a common specific biomarker. FDA, for the first time, approved a drug based on a common biomarker, instead of the organ the tumor had originated. Despite this, majority of studies still consider each cancer disease in isolation from the rest and identify the treatment options that are cancer-type specific. Hence, the critical need is to discover multi-cancer treatment options through the exploitation of cancers with similar molecular and genetic aberrations.

Mutations in several genes within the homologous recombination (HR) pathway occur in around 20%–25% of advanced prostate cancers (Marshall et al., 2019). There is accumulating evidence that depicts a considerable proportion of individuals with metastatic breast cancer are HR deficient with mutations in *BRCA1/BRCA2* genes (den Brok et al., 2017). Base excision repair (BER) pathway genes limit the ability of DNA repair in prostate cancer (PCa, henceforth) patients, which leads to an increased risk of PCa. (Mittal et al., 2012). Further, *APEX1*, which is a BER gene, has shown a compelling effect indicating an increased risk of breast cancer through a gene-gene interactivity analysis (Kim et al., 2013). In an effort to understand the effect of mismatch repair (MMR) genes in the progression of PCa, gene expression-based analysis were conducted within the cancer cell lines and in tumor specimens, which indicated a loss of *MSH2* and *MLH1* genes in different cell lines (Chen et al., 2001). The deficiency of MMR genes was observed across most of the subtypes of breast cancers with high-grade tumor-infiltrating lymphocyte counts (Cheng et al., 2020). All these findings confirmed that there were significant commonalities across breast and prostate cancers in their DNA repair pathway abnormalities that could lead to common and jointly important treatment options.

Drug repurposing strategies can be classified into drug-based and disease-based, depending on the substantial availability of data and the intent of the research (Jarada et al., 2020) (Dudley et al., 2011). Several computational approaches proposed in recent years have used both disease and drug data (Peyvandipour et al., 2018) (Sirota et al., 2011) (Chiang and Butte, 2009) (Gottlieb et al., 2011). In a systems biology approach proposed by Peyvandipour et al. (2018) a drug-disease network (DDN) was constructed by considering drug targets, disease-related genes and all signalling pathways that were then integrated with disease gene expression signatures and drug-exposure gene expression signatures to discover novel therapeutic roles for established drugs. Nafiseh et al. used a machine learning approach to find anti-similarities between drugs and disease (Saberian et al., 2019). In their approach, they used drug exposure gene expression data, disease gene expression data and the associations between FDA-approved drugs and diseases. They used a distance metric learning (DML) algorithm where disease and the associated FDA-approved drugs had smaller distances compared to drugs not associated with disease. Luo et al. (2016) proposed a novel approach that computed the similarity between drugs and diseases. In particular, they constructed a heterogeneous network consisting of drug and disease similarity networks and drug–disease interactions and then used a Bi-Random walk (BiRW) algorithm to rank the drugs (Xie et al., 2012). Hu and Agarwal. (2009) generated a disease-drug network based on extensive drug and disease gene expression profiles which was used for identifying new indications for drugs and side effects of drugs.

In this paper, we used several state-of-the-art drug repurposing approaches to determine potential drug candidates for patients with breast or prostate cancers with common specific biomarkers. More specifically, we identified drugs with potential therapeutic effects on patients with DNA repair deficiencies.

Our contribution in this study is three-fold: 1) We initially developed a data-driven approach able to enrich the study population by integrating data from biologically similar cancers and using patient subpopulations with different types of DNA repair deficiencies which will enable personalized treatment strategies. We then used an existing approach referred to as drug-disease similarity to come up with novel treatments on the integrated data by identifying drugs that may have a therapeutic effect on patients irrespective of their cancer type. 2) We revisited our previously published deep cross cancer learning approach to identify jointly important biomarkers among breast, prostate and ovarian cancers. These biomarkers were used to identify common treatment options among those cancers through network interactions-based drug repositioning. 3) We presented the associations between the proposed drug target genes and biological functions (e.g., cell cycle) and investigated the drug target genes within the HR pathway and their interactions with the proposed drugs.

## 2 Materials and methods

### 2.1 Data preparation

The variant data and the disease gene expression data for breast and prostate cancers were obtained from The Cancer Genome Atlas (TCGA). The number of samples for breast and prostate tumors were 1,091 and 495, respectively with 120 and 53 samples with adjacent normal tissues. All expression datasets were log2 transformed. We obtained the signalling pathways from Kyoto Encyclopedia of Genes Genomics (KEGG) (Kanehisa et al., 2016). The signalling pathways are represented in the form of a directed graph, where each node represents the genes (or proteins) and the associations including activation, inhibition, etc. between the genes were represented by the edges. The large scale drug-exposure gene expression data were obtained from the Connectivity Map and the Library of Integrated Network-Based Cellular Signatures (LINCS) (Subramanian et al., 2017).

**FIGURE 1:**
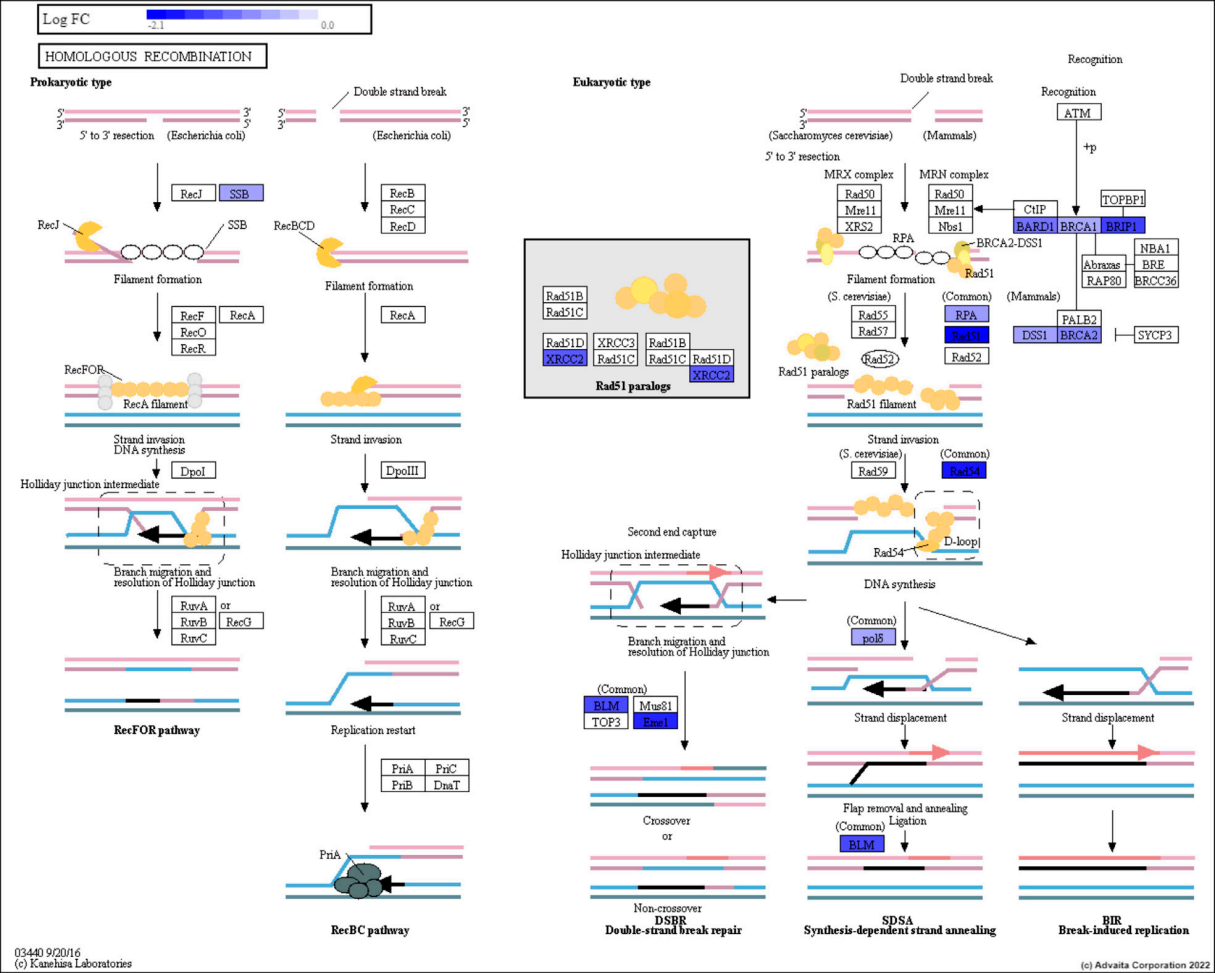
The Homologous Recombination pathway. The genes are represented in the rectangular boxes, with the shades of blue representing down-regulated genes for prostate cancer patients.

We initially identified all genes within each DNA repair pathway separately using the KEGG database. The DNA repair pathways used were: homologous recombination (HR), base excision repair (BER), mismatch repair (MMR), nucleotide excision repair (NER) and non-homologous end joining pathway (NHEJ). As an example, the set of genes (or proteins) that exist within the HR pathway can be seen in Figure 1. Using the variant data collected from TCGA, a subset of breast and prostate cancer patients with mutations in any of their DNA repair genes were identified and grouped according to their type of DNA repair deficiency. This resulted in multiple cohorts of homogeneous subpopulations with common biomarkers. Table 1 shows the distribution of the breast and prostate cancer patients within each cohort. Note that, the same patient may fall into multiple cohorts.

**TABLE 1:** The number of breast and prostate cancer patients with deficiencies in their DNA repair pathways. Note that, different types of DNA-repair deficiencies has formed several subpopulations, that were analyzed separately.

DNA repair pathway	Number of patients	
	Breast cancer	Prostate cancer
Homologous Recombination (HR)	36	14
Base Excision Repair (BER)	23	7
Mismatch Repair (MMR)	73	31
Nucleotide Excision Repair (NER)	55	23
Non-Homologous End Joining (NHEJ)	23	6
Total	210	147

Next, we identified the differentially expressed genes (DEGs) through a moderated *t*-test by comparing the tumor samples with their adjacent normal tissues on each cohort separately. The resulting *p*-values were FDR adjusted to correct for multiple comparisons. Including ovarian cancer samples would have been optimal as ovarian cancer is known to also have biological similarities with breast and prostate cancers. However, due to not having access to TCGA ovarian cancer gene expression data of *adjacent normal tissue*, we were unable to run the differential expression analysis on ovarian cancer samples in this study. An alternative approach we considered was to run experiments on ovarian cancer data collected from different data sources, however this requires extensive preprocessing due to different representation, distribution, scale, and density of data.

Our previously published deep cross cancer learning approach discussed in Section 3.3 identified jointly important biomarkers among breast, prostate, and ovarian cancers (Zhou et al., 2021). We were then able to identify drug candidates common among the three cancers using the proposed biomarkers. As this was a multi-label classification based neural network, we were able to conduct the analysis without the presence of ovarian normal tissue.

**FIGURE 2:**
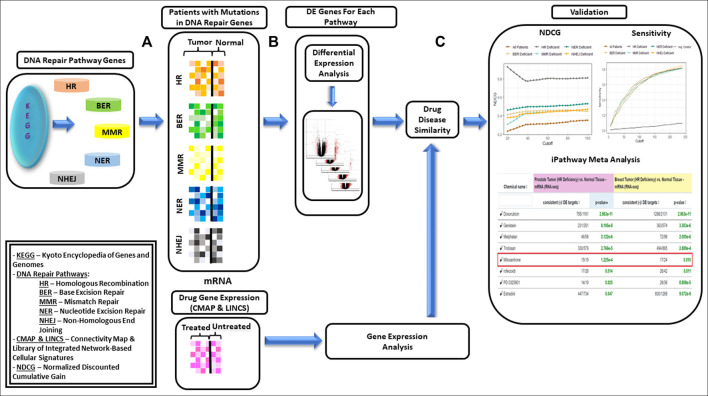
Framework proposed for data-driven drug repurposing for biologically similar cancers—**(A)**: Genes within each of the DNA repair pathways, i.e., HR (Homologous Recombination), BER (Base Excision Repair), MMR (Mismatch Repair, NER(Nucleotide Excision Repair) and NHEJ (Non-Homologous End Joining) were identified using KEGG database. Subset of breast and prostate cancer patients with mutations in DNA repair genes were identified and grouped based on DNA repair deficiency. **(B)**: Differentially expressed genes (DEGs) were identified on each cohort separately. **(C)**: Drugs for each cohort were identified using Drug-Disease Similarity. Framework was validated using NDCG (Normalized Discounted Cumulative Gain) and sensitivity scores; and network interaction analysis was used for validating the utility of the drugs.

The methodology used for data preparation described above has been shown in Figures 2A,B. Prediction of drugs using drug-disease similarity and validation shown in Figure 2C has been described in subsequent sections.

### 2.2 The prediction of drugs using drug-disease similarities

Sirota et al. (2011) proposed a systematic computational drug repurposing approach to predict novel therapeutic indications by understanding drug and disease relationships. The association between every pairing of drug and disease is represented by a similarity score ranging from +1 to −1, with +1 indicating perfect correlation and −1 indicating an opposite effect. The largest negative score representing a reverse set of changes with exposure to a drug, indicates that the drug may have a therapeutic effect on the disease.

Here, we used the preprocessed expression data as discussed in Section 2.1 for breast and prostate cancer and the drug expression signatures from CMap to calculate the similarity scores. We only considered those drugs with FDR-adjusted *p*-values less than 0.05. This shortened list was then arranged in the ascending order based on the enrichment scores. The largest negative score implied the best drug candidates with highest therapeutic effects.

In an effort to evaluate the results obtained through the drug-disease similarity model, we performed sensitivity-based validation only (SV) and calculated the normalized discounted cumulative gain (NDCG). The best strategy for analytic validation of drug repurposing is through sensitivity based validation techniques. Sensitivity and specificity based validation, although ideal, is not practical to assess the model performance due to the lack of access to true negatives (TNs) as discussed by Adam et al. (Brown and Patel, 2018). The discounted cumulative gain was constructed under the assumption that top rank drugs were more relevant and more likely to be of interest (Schuler et al., 2022). The NDGC score was calculated as follows:

$$DCG=\overset{p}{\underset{i=1}{\sum }}\frac{2^{{\text{rel}}_{i}-1}}{\log_{2}\left(i+1\right)}$$ (1)

$$IDCG=\overset{\left|REL_{p}\right|}{\underset{i=1}{\sum }}\frac{2^{{\text{rel}}_{i}-1}}{\log_{2}\left(i+1\right)}$$ (2)

NDGC=DCG/IDCG (3)

where *i* is the rank of the drug of interest, up to rank *p*, and *rel*_*i*_ denotes the relevance of the drug to the indication, 0 indicating non-relevance and 1 indicating relevance, *REL*_*p*_ is the list of associated drugs in the set up to a cutoff position of *p*, and |*REL*_*p*_| is the cardinality of the list.

### 2.3 The validation of proposed drugs using network interactions

**FIGURE 3:**
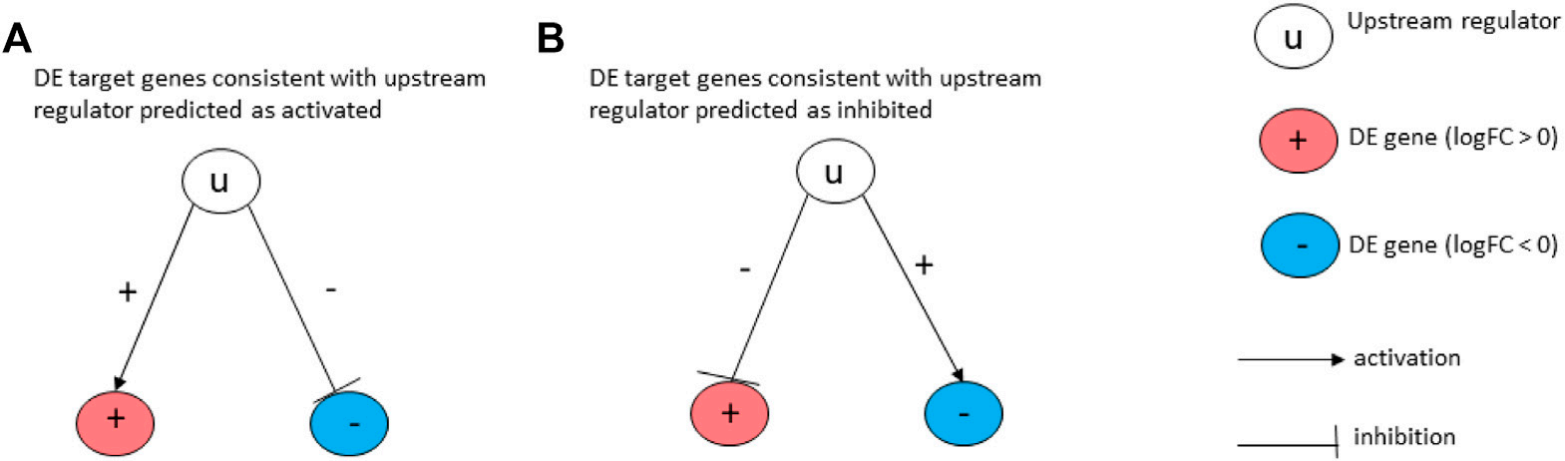
Target genes consistent with the hypothesis considered: In **(A)**, the signs of the DE genes shown in red (+) and blue (−) match the signs of their respective incoming edges, suggesting that the upstream regulator u is activated. In **(B)**, the signs of the DE genes shown in red (+) and blue (−) are opposite to the signs of their edges, suggesting that the upstream regulator u is inhibited.

Here, we used a drug repurposing analysis module to identify FDA-approved drugs that could be used to revert a given pattern of gene expression changes caused by a disease. The prediction of upstream Chemicals, Drugs, Toxicants (CDTs) is based on two types of information: 1) the enrichment of differentially expressed genes from the experiment and 2) a network of interactions from the Advaita Knowledge Base (AKB v2006). The network is a directed graph in which the source node represents either a chemical substance or compound, a drug, or a toxicant. The edges represent known effects that these CDTs have on various genes. A signed edge in this graph consists of a source CDT, a target gene, and a sign to indicate the type of effect: activation (+) or inhibition (−). To generate the network, the analysis selects only those edges observed in the literature with at least a medium confidence. The analysis considers two hypotheses: HA: The upstream regulator is activated in the condition studied. HI: The upstream regulator is inhibited in the condition studied. The set of genes from National Center for Biotechnology Information (NCBI) Gene database is divided into many subsets by the analysis based on the measurements from the experiment and the definitions shown in Figure 3. The (+) sign in the figure indicates up-regulated genes while (−) sign indicates down-regulated genes. If a gene has at least one incoming edge, then it is considered as a target gene in the network. The gene g is consistent with hypothesis HA if there is an incoming edge e and if sign(g) = sign(e). This implies that when upstream regulator is activated, the signal is an activation and gene is up-regulated or signal is an inhibition, and the gene is down-regulated. (see Figure 3A). The gene g is consistent with hypothesis HI if there is an incoming edge e and if sign(g) does not match sign(e). This implies that when upstream regulator is inhibited the signal is inhibition and gene is up-regulated or signal is activation and gene is down-regulated. (see Figure 3B).

Herein, we focused on drugs that could reverse the changes induced by the disease. For this purpose, we hypothesized that the disease is considered as a state in which the changes are associated with the absence of a drug. Given the interactions between a specific drug A and its downstream DE genes, the Z-score was computed as follows:

$$z\left(A\right)=\frac{\sum_{e,g}w\left(g\right)\text{.}s\left(e\right).s\left(g\right)}{\sqrt{\sum {\left|w\left(g\right)\right|}^{2}}}\;$$ (4)

where *s*(*e*) represents the type of the edge (−1 for inhibition and +1 for activation), *s*(*g*) is the sign of expression change of the gene (−1 for down-regulated and +1 for up-regulated), and *w*(*g*) the confidence score of the edge *g*. The Z-score *p*-value for each drug was then calculated by mapping the z-score on a *p*-value using the normal distribution. (Draghici et al., 2020).

Note that, the drugs identified through drug-disase similarities as discussed in Section 2.2, though powerful, do not consider the network of interactions between drugs and their associated downstream genes. On the other hand, the network interactions as discussed in this section may still not be able to detect all significant drugs as only direct interactions between drug and disease is considered, rather than investigating indirect interactions due to co-expressions of genes. Hence in order to identify drugs with high therapeutic effects, we relied on the intersecting drugs among multiple approaches.

## 3 Results

### 3.1 Drug-disease similarity results

The results obtained through the drug-disease similarity analysis are shown in Table 2. Initially, all breast and prostate cancer patients were included in the analysis which resulted in a list of drugs presented in the first column of the table (see column: All Patients). In essence, a good repurposing approach on a truly homogeneous data should place the already FDA-approved drugs (i.e., the gold standard) at the very top of the list for that particular disease. Note that, since we focussed on multiple biologically similar diseases, we expected to see drugs approved for either or both of the conditions at the very top of the list.

**TABLE 2:** The list of top ranked drugs identified through the drug-disease score analysis for subsets of patients with different types of DNA repair deficiencies. The cells highlighted in green, grey, blue and pink are the FDA-approved drugs, investigational drugs for breast and prostate cancers, investigational drugs for prostate cancer, and investigational drugs for breast cancer, respectively along with their respective similarity scores that was calculated. Results demonstrated that although there are certain drugs that are common across subpopulations, the top ranked drugs differed between different DNA-repair pathways. Hence, the identification of biomarkers associated with a specific subpopulation can change the course of treatment and enable personalized treatment strategies among individuals.

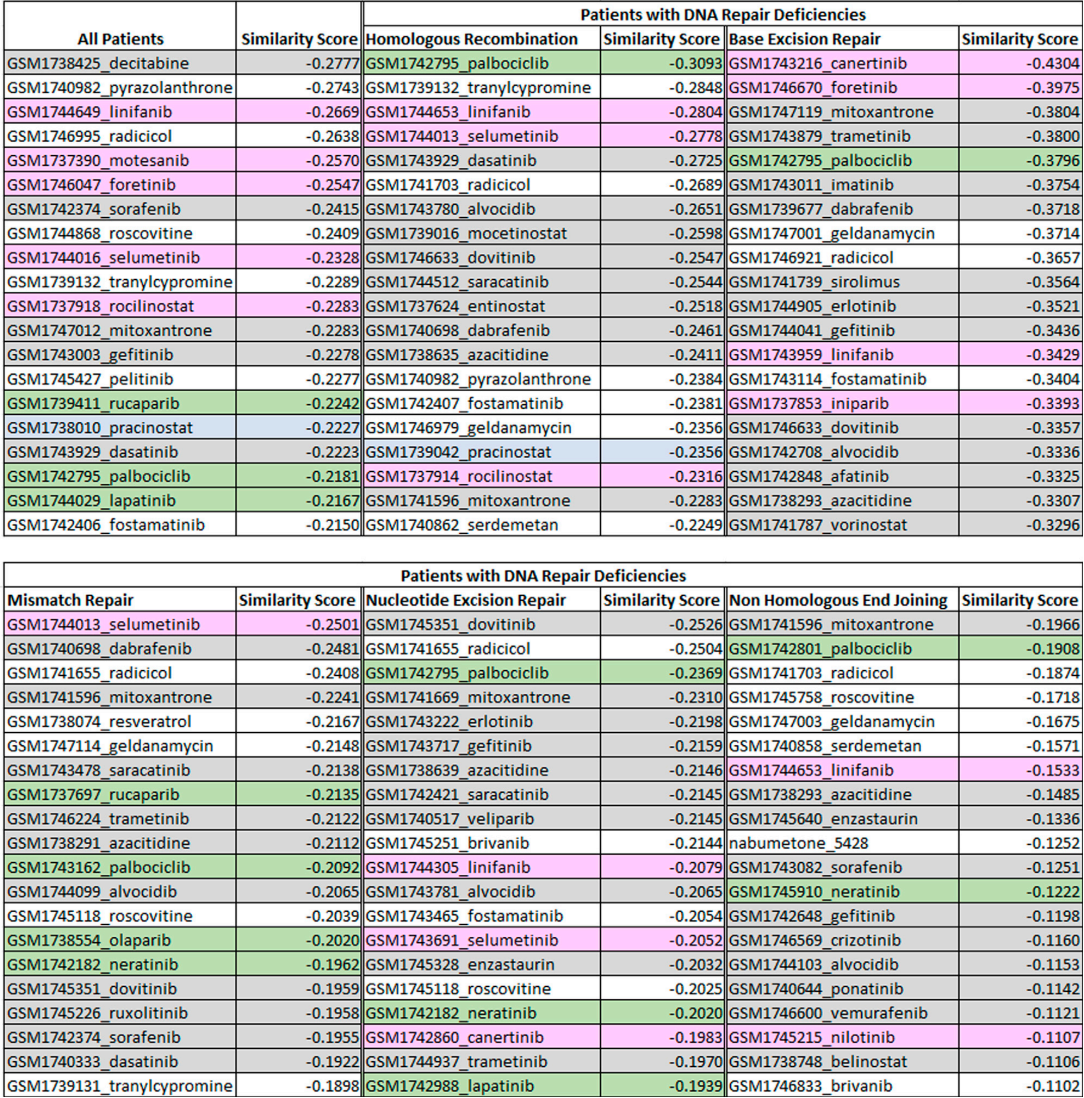

Results showed that six investigational drugs (two of which are under investigation for breast and prostate cancers, and four of which are under investigation for breast cancer only) and no FDA-approved drugs appeared within the top 10 ranked drugs. Cancer being a heterogeneous disease with large genetic diversity even between tumors of the same cancer types, it is common for the patients to have significant differences between their molecular profiles (Arslanturk et al., 2020). Our results clearly showed that the data needed to be further refined to identify more homogeneous subpopulations for more optimal and targeted treatment decisions. Hence, as the next step, we investigated potential treatment options based on common biomarkers, specifically for patients with aberrations in genes within different DNA repair mechanisms. Results showed Palbociclib, an endocrine-based chemotherapeutic agent approved for treating HER2-negative and HR-positive advanced or metastatic breast cancers (McCain, 2015) (Walker et al., 2016) (Beaver et al., 2015), appeared at the top of the list for patients with HR-deficiencies. Results further suggested that tranylcypromine, a monoamine oxidase inhibitor, mainly approved for the treatment of major depressive episodes without melancholia (Ricken et al., 2017), showed promise as a multi-cancer treatment, specifically for breast and prostate cancers. The top ranked drugs further consisted of several chemotherapy drugs including linifanib, selumetinib and dasatinib. The top ranked drugs for all other DNA repair deficient patients are listed in Table 2. A detailed description of all the top ranked drugs for each pathway along with their clinical relevance is reported in the discussion section of the paper.

**FIGURE 4:**
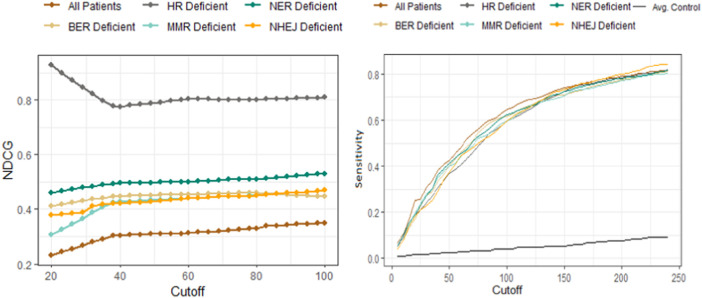
Performance comparison of the drug-disease similarity model on DNA-repair deficient patient subpopulations using NDCG (left) and sensitivity analysis (right). The NDCG/sensitivity values (vertical axes) of all drug–indication associations using different DNA repair deficient subpopulations are shown according to different cutoff values (horizontal axis). The NDCG results clearly demonstrate that the HR-deficient subpopulations result in drugs that are clinically more relevant with more FDA-approved/investigational drugs compared with other DNA-repair pathway deficiencies. The plot has further shown that identifying homogeneous subpopulations through common biomarkers result in better performances when compared to all patients combined. The sensitivity values demonstrate that the list of breast/prostate cancer drugs retrieved for all cutoff levels are clinically relevant and indicates an overall better performance relative to random controls (shown as the black curve).

The sensitivity and NDCG scores of the proposed drugs are shown in Figure 4. The sensitivity values of all drug-disease associations for different subsets of patients based on their types of DNA repair deficiencies were compared with several random control runs. The sensitivity values were reported for different rank/cutoff levels. The SV results as shown in Figure 4B demonstrates that the list of drugs retrieved for all cutoff levels for breast and prostate cancer patients were clinically relevant and indicated an overall better performance relative to random controls. The NDCG scores as shown in Figure 4A show that the identification of homogeneous sub populations with common biomarkers resulted in drugs that were clinically more relevant with more FDA-approved/investigational drugs appearing at the very top of the list when compared with all patients combined. Results further showed that drugs proposed for patients with aberrations in their HR pathway outperformed all other pathways. This is mainly due to hormone driven cancers’ significant molecular similarities within HR pathways (Toh and Ngeow, 2021) (Watkins et al., 2014). Less is known about the similarities between those cancers in other DNA-repair pathways.

### 3.2 Drugs proposed through network interactions

The drugs proposed through network interactions using iPathwayGuide (Advaita) are listed in Table 3. Note that, this table includes only the drugs that have a significant therapeutic effect (*p* < 0.05) on both breast and prostate cancers. The number of DE genes that would be reverted by each drug is listed. For instance, the 15/19 notation next to mitoxantrone demonstrates that there were 19 downstream genes that mitoxantrone is interacting with that were DE for prostate cancer (vs. adjacent normal tissue), 15 of which were consistent with our hypothesis as described in Section 2.3.

**TABLE 3:** The top eight drugs proposed for repurposing using the network interactions approach. The table shows the *p*-values (sorted based on the prostate tumor vs. adjacent normal tissue experiment), as well as the number of DE genes that would be reverted by each drug (i.e., the number of genes consistent with the hypothesis) for patients with HR−deficiencies. Doxorubicin slows or stops the growth of cancer cells, and is used to treat certain neoplastic conditions such as acute lymphoblastic leukemia, soft tissue and bone sarcomas, breast carcinoma and ovarian carcinoma. Genistein is currently under clinical trials for the treatment of prostate cancer. Melphalan and Estradiol are also among drugs used to treat certain cancers. Mitoxantrone is highlighted as a promising drug candidate as it appears to be a top drug using both network interactions and drug-disease similarity scores.

Chemical name	Prostate tumor (HR deficiency) vs. Normal tissue - mRNA (RNA-seq)		Breast tumor (HR deficiency) vs. Normal tissue - mRNA (RNA-seq)	
	Consistent (-)/DE targets	*p*-value	Consistent (-)/DE targets	*p*-value
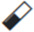 Doxorubicin	785/1161	2.863e-11	1288/2101	2.863e-11
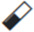 Genistein	231/351	8.195e-8	363/574	3.503e-6
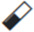 Melphalan	46/58	2.122e-6	72/98	2.005e-6
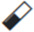 Triclosan	330/579	2.766e-5	494/865	2.889e-4
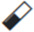 Mitoxantrone	15/19	1.225e-4	17/24	0.01
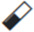 rofecoxib	17/26	0.014	26/42	0.011
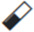 PD 0325901	14/19	0.025	28/36	8.808e-5
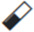 Estradiol	447/734	0.047	830/1268	9.072e-9

**FIGURE 5:**
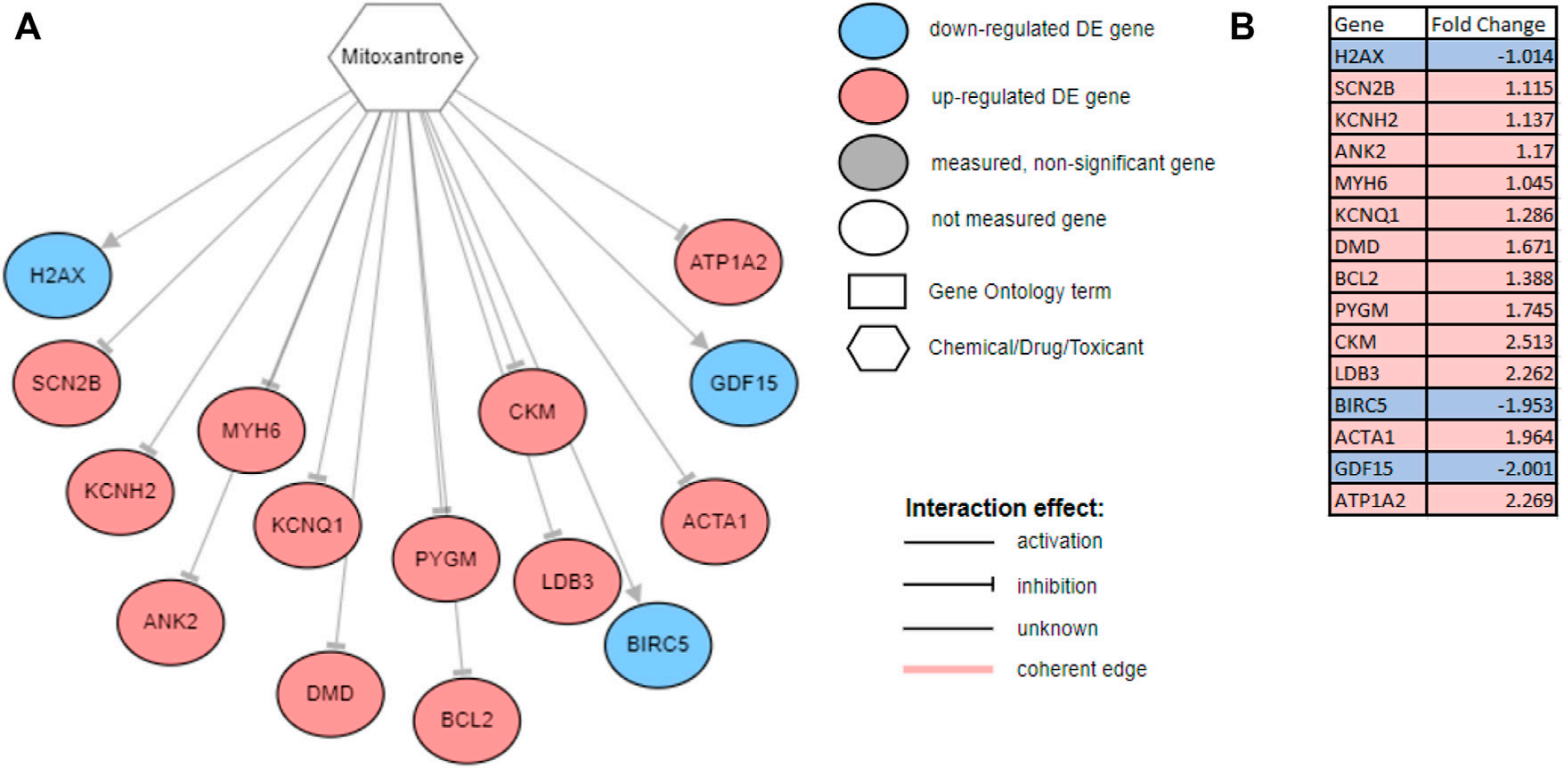
**(A)** The mechanism through which Mitoxantone act on the genes measured to be DE for prostate cancer. Note that, out of the 19 downstream DE genes that Mitoxantone is interacting with, 15 were consistent with the hypothesis, i.e., the drug was able to revert the expression changes caused by disease 15 out of 19 times. All 15 genes were shown on the figure with three down-regulated genes (blue circles) being activated, and 12 up-regulated genes (red circles) being inhibited with the exposure of the drug. **(B)** Fold changes reported for cell lines treated with Mitoxantrone. The upregulated genes are highlighted in red, and the downregulated genes are highlighted in blue.

The SV and NDCG are metrics used to evaluate the drug repurposing models’ ability to identify clinically relevant treatment options. In order to validate the utility of the drugs proposed, we investigated the mechanisms through which the drugs act on genes measured to be DE for the disease studied. Figure 5 generated using network interactions shows the mechanisms of mitoxantrone on the DE genes for prostate cancer. Mitoxantrone was able to activate the down-regulated genes and inhibit the up-regulated genes 15 out of 19 times (*p* < 1.225e-4) as described in Section 2.3 In an effort to confirm the changes in the downstream genes, we have reported the fold-changes of those genes using cell lines treated with Mitoxantrone as shown in Figure 5B. The upregulated genes are highlighted in red, and the downregulated genes are highlighted in blue.

### 3.3 Drugs proposed using novel biomarkers discovered using cross cancer learning approach

**FIGURE 6:**
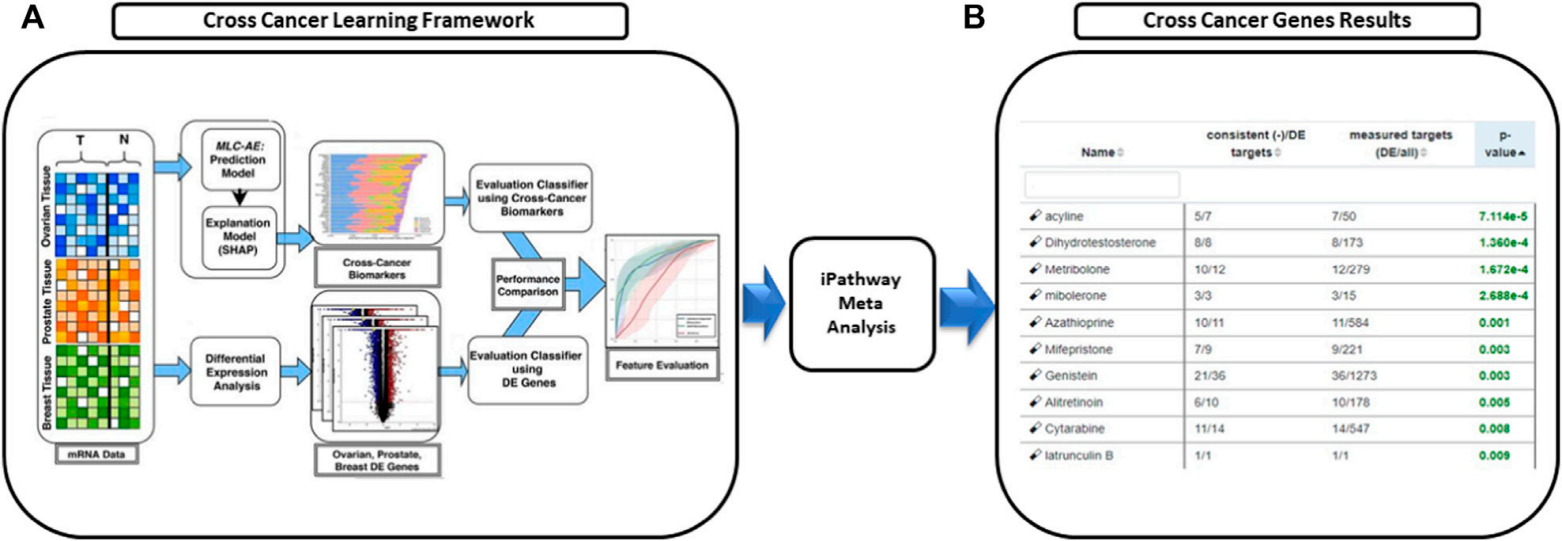
Drugs proposed using cross cancer genes. **(A)** Breast, prostate and ovarian cancer expression data was used to predict the tissue type and the disease type using multi-label classification—auto encoder (MLC-AE). SHAP Explanation model was used to identify the contribution of each gene towards the prediction using SHAP values that rank the genes. **(B)** Network interaction analysis was used to perform meta analysis and predict novel drugs.

**FIGURE 7:**
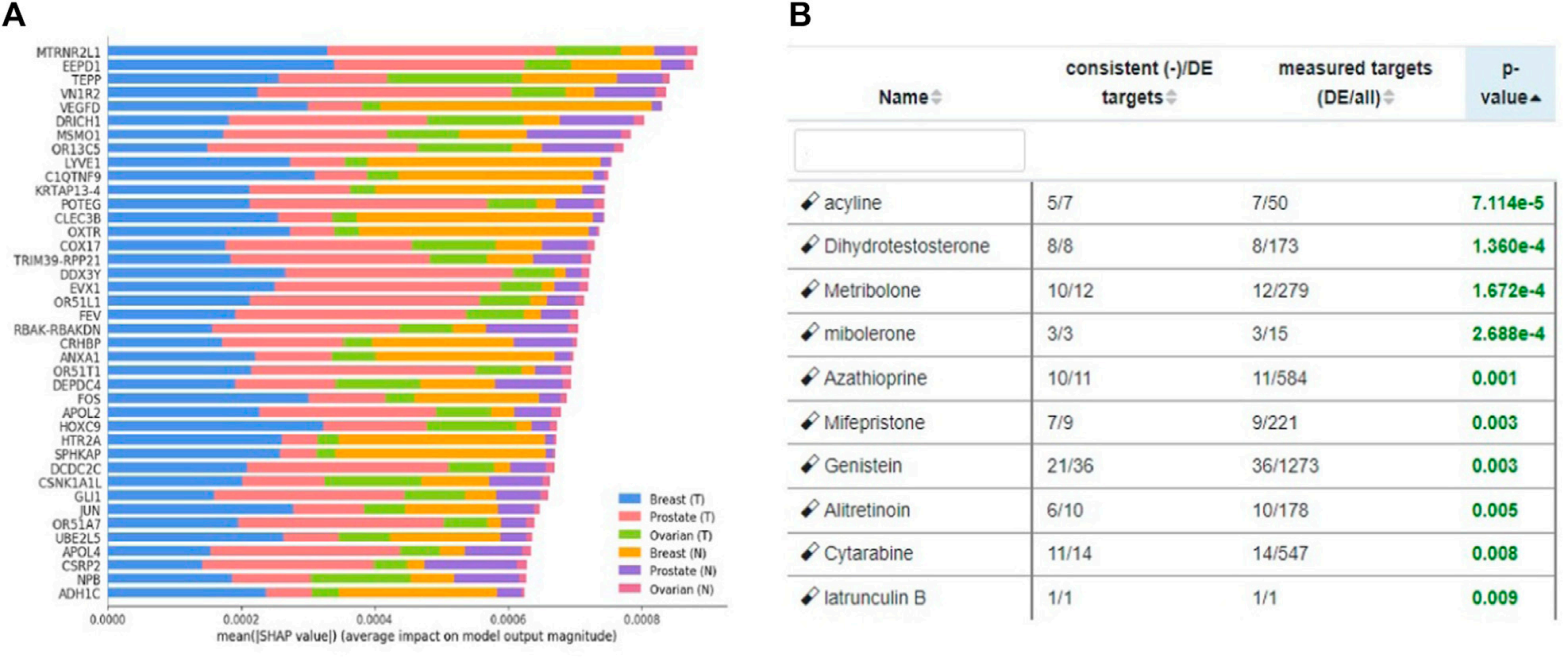
**(A)** Significant genes identified using SHAP based on contribution scores for all three tissues (breast, prostate and ovary). (T) Denotes solid tumor and (N) denotes solid normal tissue. Figure utilized from Zhou et al. (2021) **(B)** Top eight drugs proposed for repurposing using cross cancer genes.

We utilized our previously published approach that discovered jointly important novel biomarkers across breast, prostate and ovarian cancers through a data-driven, deep learning approach referred to as cross-cancer learning (Zhou et al., 2021). This approach exploited patient data from multiple cancers to discover prostate cancer biomarkers and jointly important biomarkers across breast, prostate and ovarian cancers by leveraging pathological and molecular similarities in their DNA repair pathways. Different cancers share common genomic instabilities. Exploring cancers having similarities can help discover previously unknown biomarkers and pathways. In addition, this helps in alleviating the problem of limited patient samples availability and underestimation of various genes previously not known to be involved. This cross cancer learning framework utilized a multi-label classification autoencoder (MLC-AE) that used lower dimensional latent representation of the mRNA gene expression profiles to predict the tissue type (breast, prostate, ovarian) and the disease state (solid tumor vs. adjacent normal tissue) as separate output layers. To explain and interpret the MLC-AE model, SHapley Additive exPlanations (SHAP) was used. This method uses SHAP values to extract feature importance across three cancers. SHAP method used each feature to calculate the change in performance in the presence and absence of each feature. The features whose absence lead to reduction in the performance were given the highest score. The cross cancer framework has been shown in Figure 6. Figure 7 A shows the most significant genes based on their contribution towards prediction using breast, prostate, and ovarian tissues. The biomarkers discovered using this approach were further used to find disrupted pathways using the impact analysis. The drugs identified using cross cancer genes are listed in Figure 7B and are discussed in detail in the Discussion section.

In order to validate our results further, additional experiments were conducted using the cell lines obtained from CMap. Table 4 shows the fold changes that were calculated using the cell lines treated with the drugs shown on each column. Specifically, the drugs investigated were Genistein, Mitoxantrone, Palbociclib, Tranylcypromine, Linifanib and Selumetinib. Threshold parameters used for the analysis were an absolute fold-change greater than 0.6 and false discovery rate (FDR) adjusted *p*-value less than 0.05. All genes presented in the table are differentially expressed, with the genes associated with DNA repair pathways being color-coded. Specifically, the red, yellow, green, blue, and gray colors represent significant changes in the genes associated with BER, HR, MMR, NER and a combination of multiple DNA repair pathways, respectively.

**TABLE 4:** This table shows the expression changes of genes when the drugs that were found to be significant in our analysis were administered. Red, yellow, green, blue, and gray colors represent significant changes in the genes associated with BER, HR, MMR, NER, and a combination of multiple DNA repair pathways, respectively.

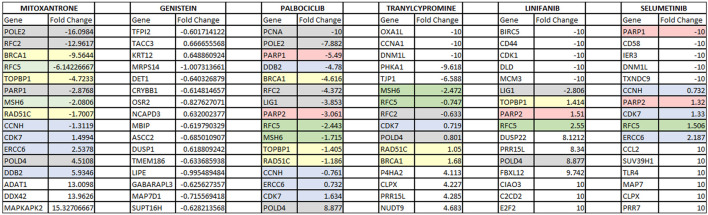

Note that there are no differentially expressed genes involved in the DNA repair process for Genistein. However, expression changes obtained from CMap includes an arbitrary selection of patients and is not filtered based on homogeneous subpopulations identified through specific DNA repair deficiencies. Instead, our proposed drug candidates have been derived by filtering a list of patients with specific types of DNA repair deficiencies, and therefore, is a preprocessed dataset with a more homogenous population than the CMap patient set. Although this could explain the lack of gene changes in DNA repair pathways when Genistein is administered, additional analyses would be required to further confirm the therapeutic effect of this drug.

**FIGURE 8:**
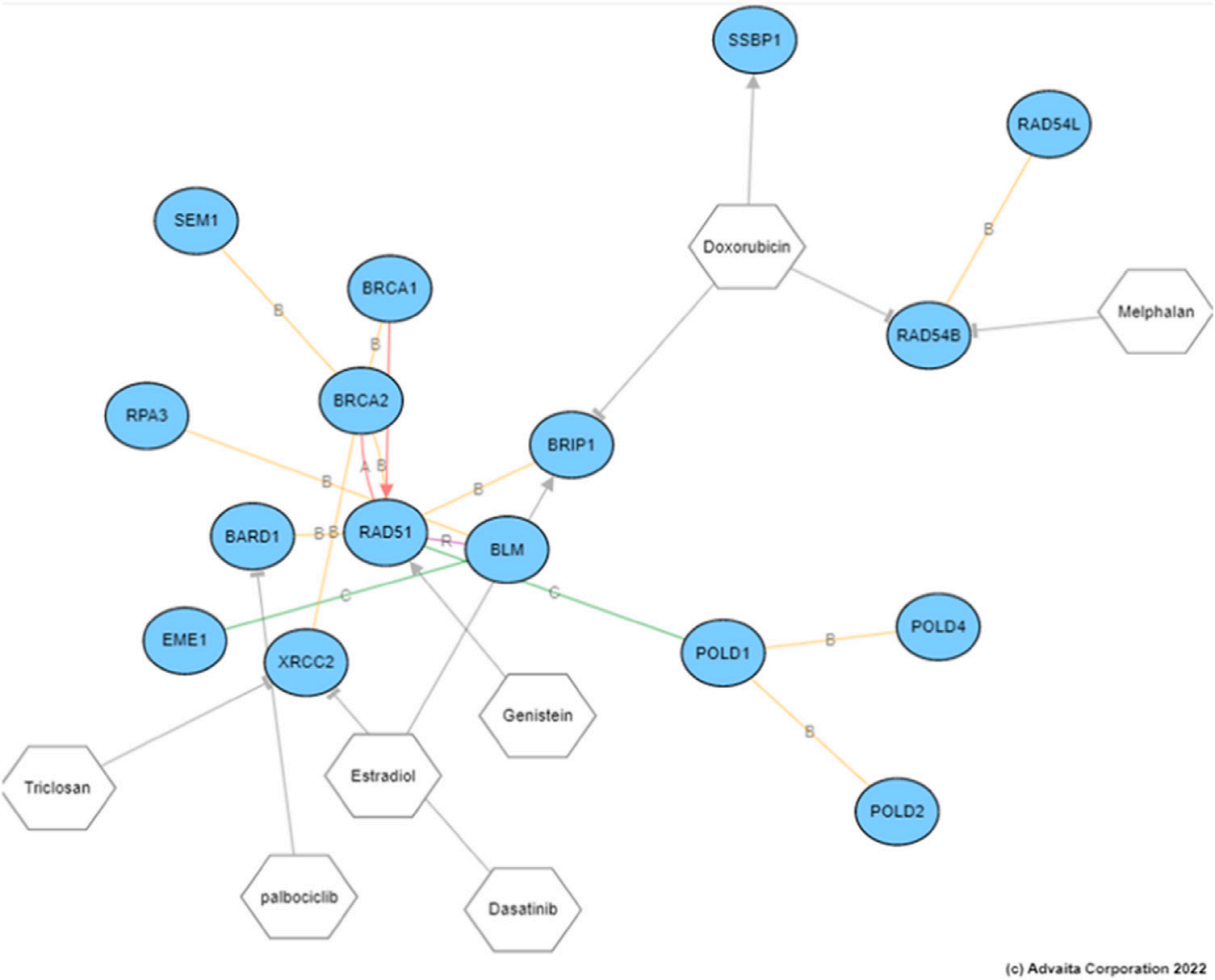
The drug target genes within the HR pathway and their interactions with the proposed drugs. The differential expression analysis here were conducted on patients with breast cancer with HR deficiencies vs. adjacent normal tissue.

In order to understand the effect of our proposed drugs on the nodes within the HR pathway, we explored the drug-gene interactions. The results are shown in Figure 8. The differentially expressed genes highlighted in this figure are based on patients with HR deficient breast cancer vs. adjacent normal tissue. This figure clearly shows that several HR genes are indeed drug targets and our proposed drugs are indeed interacting with such genes.

In summary, our results showed several promising drug candidates including Mitoxantrone, Palbociclib and Genistein for multi-cancer treatment as supported by multiple approaches. Mitoxantrone appeared to be a top drug using drug-disease similarity scores and network interactions approaches, and Genistein appeared to be a top drug using cross-cancer biomarkers and network interactions.

**FIGURE 9:**
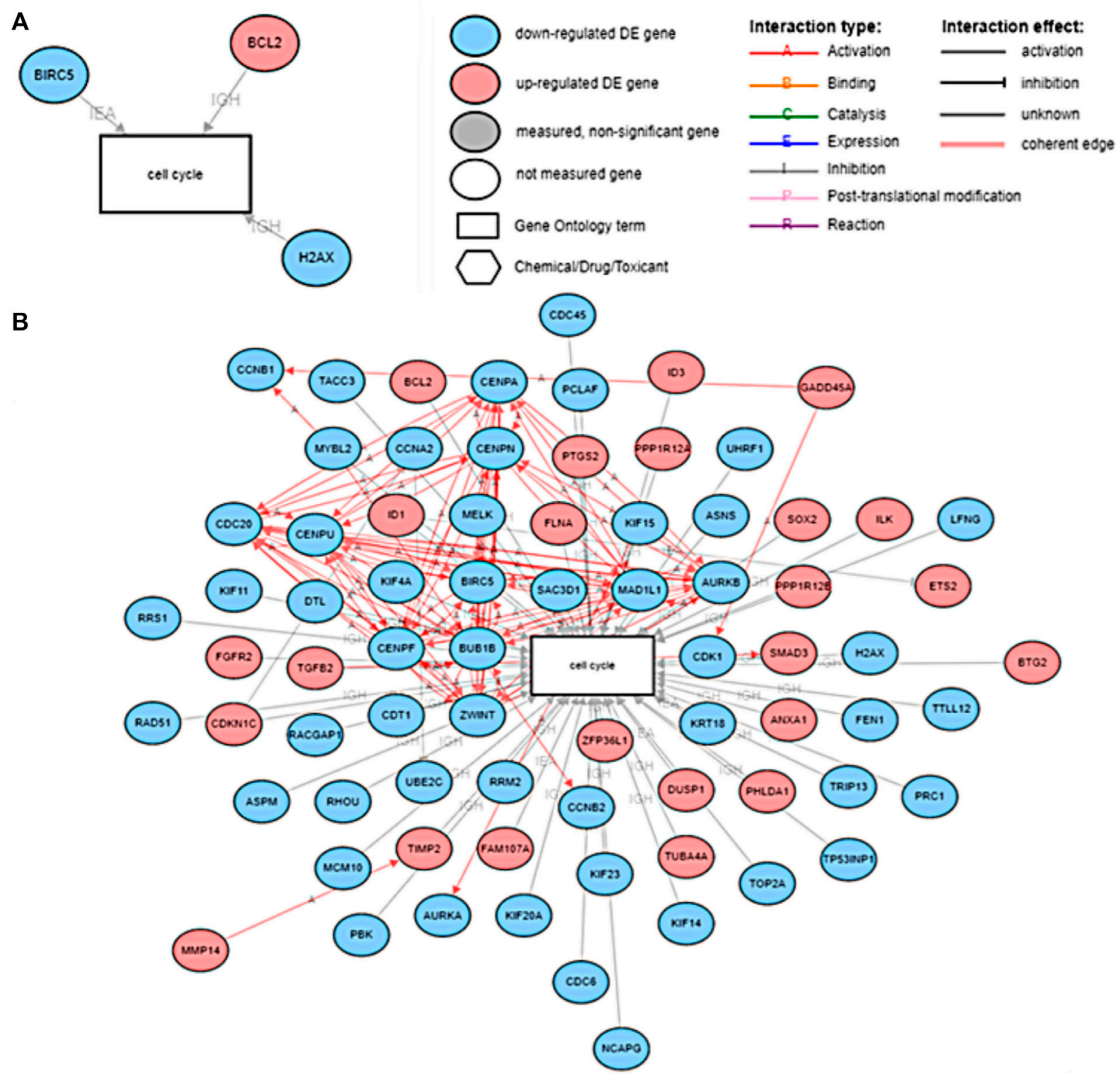
**(A)** The DE genes of prostate cancer associated with the cell cycle downstream of Mitoxantrone and **(B)** The DE genes of prostate cancer associated with the cell cycle downstream of Genistein.

Experiments conducted by Tang et al. (2018) and Siddiqui et al. (2021) suggest that genistein and mitoxantrone in combination with other drugs can influence the cell cycle of the cancer cells. In order to understand the effects of these drugs on cell-cycle, we ran an experiment on iPathwayGuide, to understand the associations between the downstream genes of Genistein and Mitoxantrone and their associations with biological processes including the cell cycle. Results are presented in Figure 9.

## 4 Discussion

DNA damage is not uncommon and results in tens of thousands of damages everyday (Jackson and Bartek, 2009; O’Connor, 2015). This genomic instability is the key feature of carcinogenesis. DNA damage response (DDR) collectively refers to all the mechanisms that are responsible for the DNA damage repair. O’Connor. (2015) discussed targeted therapies based on DNA damage response of patients to tailor targeted therapy. They further mentioned various drugs under clinical trials for different types of cancers targeting DNA repair pathways.

Homologous Recombination is responsible for the repair of DNA double stranded breaks (DSBs) during G2/M phase (Saleh-Gohari and Helleday, 2004). Li and Heyer. (2008); Al-Mugotir et al. (2021) showed that doxorubicin, and quinacrine, along with mitoxantrone were effective in HR deficient cells by recruiting *RAD52* to repair sites of DNA damage.

Table 2 shows the drugs that were identified using drug-disease score analysis for the subset of patients who had deficiencies in their DNA repair pathways for prostate cancer and breast cancer. In the list of drugs identified for HR pathway, palbociclib came as significant. Palbociclib is approved for HER2-negative and HR-positive advanced or metastatic breast cancer. It is known that *BRCA1* and *BRCA2* mutations are involved in the HR deficiency. Hence, this could be a promising drug for the prostate cancer patients who at present do not respond to the current treatment. The network interactions approach shown in Table 3 came up with interesting set of drugs. Studies have shown that Genistein affects cell cycle during G2/M phase (Zhang et al., 2013). Genistein inhibits protein-tyrosine kinase and topoisomerase-II (DNA topoisomerases, type II) and is under investigation as an anti-cancer agent. *In vivo* experiments carried out by Tang et al. (2018). have showed that Genistein when combined with AG1024 (a tyrosine kinase inhibitor) led to a decrease in tumor size in prostate cancer patients. Genistein suppressed the homologous recombination (HR) and the non-homologous end joining (NHEJ) pathways by inhibiting the expression of *Rad51* and *Ku70* (Tang et al., 2018). Genistein, an isoflavone found in soy products and an integral part of the Asian diet, was found to be effective against various cancers and responsible for lowering the prostate and breast cancer rates in Asian countries. It inhibited the cell cycle proliferation and induces apoptosis. (Banerjee et al., 2008). Khan et al. (2021) described the emerging role of natural products in cancer treatment. Among them, soy isoflavones, were reported to target *BRCA* histones for repair. Through their *in vivo* experiments, Fan et al. (2006) found that genistein along with indoole-3-carbinol targeted both *BRCA1* and *BRCA2* genes in breast and prostate cancer cells. This research is useful in suggesting that natural products can be potential therapeutics for cancer treatment.

Al-Mugotir et al. (2021) listed mitoxantrone as a potential drug for clinical use targeting Topoisomerase II. Siddiqui et al. (2021) showed that mitoxantrone along with imatinib could be used to suppress apoptosis. Their research specifically targeted treatment-resistant HR-proficient cancers. *RAD52*, a protein involved in the HR pathway, was found to be differentially expressed in BRCA-deficient cells. The changes in the expression of the gene *RAD52* is associated with HR activity and hence can affect the way cancer can be treated (Nogueira et al., 2019), (Lok and Powell, 2012). Al-Mugotir et al. (2021) reported that *RAD52* could be a potential target for the HR deficient cancers and further showed the effectiveness of mitoxantrone on such cancers. These findings further strengthen our proposed results of mitoxantrone as a potential candidate for patients with mutations in their HR repair pathways.

In a study conducted on COX-2 inhibitors and breast cancer patients between 1998–2004, it was shown that rofecoxib had the highest percentage (71%, *p* < 0.01) of breast cancer reduction as compared to other drugs including ibuprofen (63%) and 325 mg aspirin (49%). (Harris et al., 2014).

Estradiol is already in use for breast and prostate cancers for palliation therapy.

Figure 7B shows the drugs that were listed as siginificant for the novel biomarkers discovered using cross cancer learning approach by Zhou et al. (2021) Acyline showed as significant drug in our table. In a study conducted by Sofikerim et al. (2007) to find the hormonal predictors of the prostate cancer, follicle-stimulating hormone (FSH) was found to be significantly higher in patients with prostate cancer. Crawford et al. (2017) discussed about evidences of high levels of FSH in the advanced and metastatic prostate cancer. Christenson and Antonarakis. (2018) discussed the use of gonadotropin-releasing hormone (GnRH) agonists to inhibit FSH levels as an initial step once prostate cancer turns metastatic. In the first experiment conducted on humans, Herbst et al., (2002) found that acyline, a novel GnRH antagonist was found to suppress FSH levels. They discussed the use of acyline as a probable prostate cancer drug. O’Toole et al. (2007) discussed the potential use of acyline for breast cancer and prostate cancer. Limonta et al. (2012) discussed GnRH agonists decreasing the tumor growth and proliferation in prostate, ovarian and breast cancers. Genistein, which came up as significant for HR-deficient patients earlier, was listed as significant for cross cancer genes as well and has been discussed earlier.

Currently, there is a strong evidence that the biologically similar cancers have the same underlying genetic aberrations (Risbridger et al., 2010). Hence, providing jointly important treatments could drastically reduce the time invested in development of novel drugs as well as repurposing drugs for diseases separately. Our study exploited the prostate cancer and breast cancer patients with deficiencies in their DNA-repair pathways. There is not clear understanding of DNA repair pathways (excluding HR pathway) involved in the breast and prostate cancer, and hence may require further study. There is a strong evidence that a subset of prostate and breast cancer patients have deficiencies in their HR pathways. The drugs proposed using our approach for this pool of patients have strong evidence from literature and show strong promise.

## 5 Conclusion

DNA repair pathways are responsible for maintaining the genome stability by performing various mechanisms to reverse the damage caused. Failure to do so may result in various diseases, including cancer. Most malignancies arise from mutations caused by damage to the DNA that was not repaired. While some patients respond to treatments, a subset of patients do not respond to the standard treatments. This clearly concludes that there is heterogeneity within the same type of cancer that needs to be further refined.

In this paper, we identified commonalities and differences among multiple cancers by leveraging the abnormalities within the DNA repair pathways to identify potential drugs through repurposing. Often, a specific drug repurposing approach may not always provide optimal results due to its limitations. Hence, we employed multiple approaches and provided treatment options that were intersecting between the approaches.

Our multi cancer treatment model 1) integrated subsets of patients with common biomarkers in their DNA repair pathways and 2) provided promising drug candidates for patients with different DNA repair deficiencies. The results of the proposed framework can be further utilized as a personalized medicine option for patients who do not respond to regular and organ specific treatment options.

## Data Availability

Publicly available datasets were analyzed in this study. This data can be found here: http://cancergenome.nih.gov.
